# P-1612. Clinical Outcomes and Socio-demographic Disparities among COVID-19 Patients with Autoimmune Diseases

**DOI:** 10.1093/ofid/ofaf695.1790

**Published:** 2026-01-11

**Authors:** Jessica Ngo, Dwayne M Baxa

**Affiliations:** Oakland University William Beaumont SOM, Rochester, MI; Oakland University William Beaumont School of Medicine, Rochester, Michigan

## Abstract

**Background:**

COVID-19, caused by SARS-CoV-2, has disproportionately affected racial/ethnic minorities and immunocompromised individuals. Black, Hispanic, and Asian Americans face greater risks of COVID-19 positivity and ICU admission. COVID-19 shares immunologic features with autoimmune diseases. This study examines differences in COVID-19 outcomes between hospitalized patients with and without autoimmune diseases.

**Figure:**
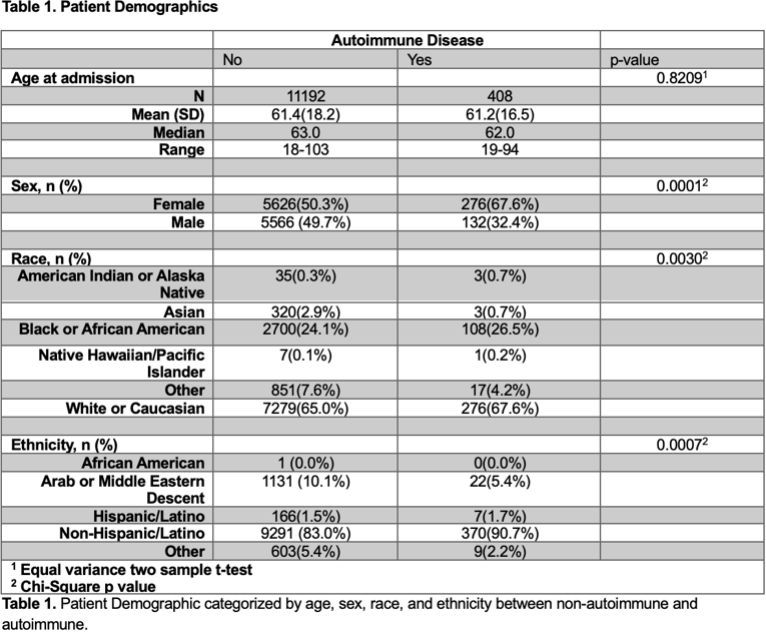

**Figure:**
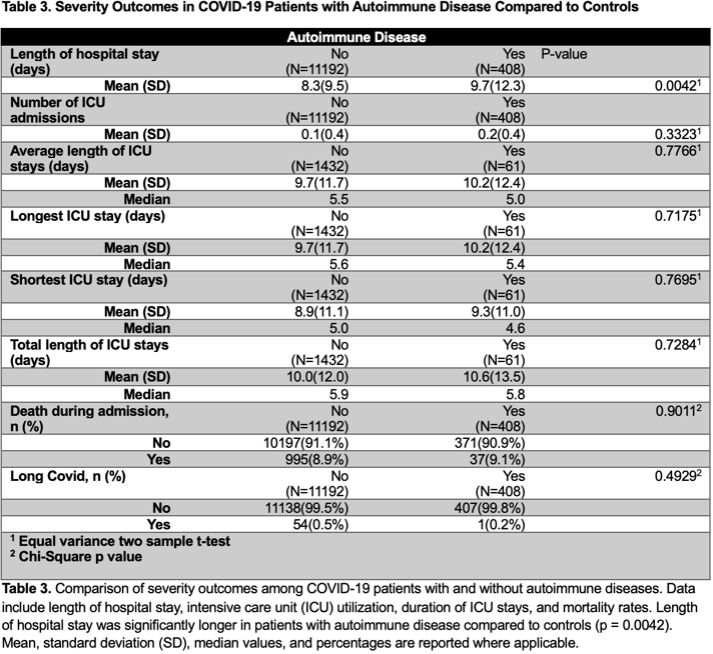

**Methods:**

We conducted a retrospective study of data from a Metro Detroit multi-center health system from Dec 2019 - Nov 2021. Demographic data, length of stay (LOS), ICU admission, diagnosis, and mortality were analyzed. Patients were stratified by autoimmune status. Chi-square and t-tests were used for statistical analyses.

**Figure:**
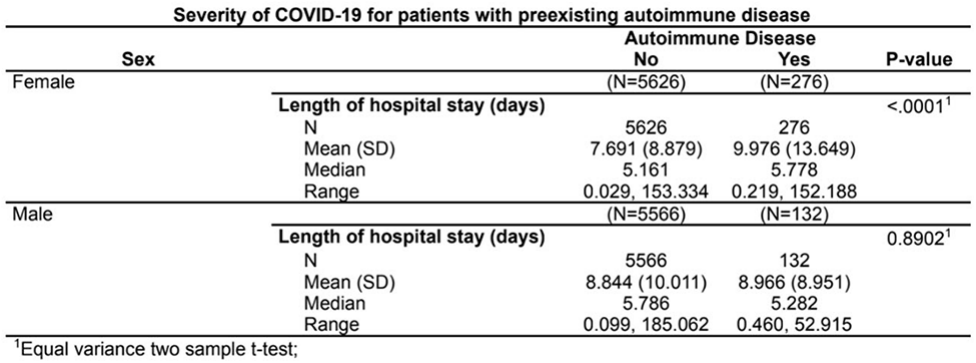

**Results:**

Of 11,600 hospitalized COVID patients, 408 had preexisting autoimmune diseases. The non-autoimmune cohort was 50% female; the autoimmune group 67.6% female (p< 0.0001) and White (67.6%). Asians and those of Arab/Middle Eastern descent were more prevalent in the non-autoimmune group (2.9% and 10.1% respectively). A slightly higher proportion of females had autoimmune conditions across racial groups (p=0.0328): Black (30.4% vs 27.6%), White (63.8% vs 62.5%). While more Asians and Native American/Alaskan were non-autoimmune (2.8% and 0.4% respectively). Autoimmune females had longer LOS (mean 9.98 vs 7.69 days, p< 0.0001) with no difference in mortality between the groups. Mean LOS for Black (10.87 vs 8.49; p=0.0269)) and White (9.40 vs 8.14; p=0.0235) patients was greater in the autoimmune group. Common autoimmune conditions included rheumatoid arthritis (26.5%), systemic lupus erythematosus (12.7%), type 1 diabetes (8.7%), and sarcoidosis (14%).

**Conclusion:**

Autoimmune patients hospitalized with COVID-19 experienced longer hospital stays and distinct demographic patterns, reaffirming the burden of autoimmune disease among women and those with comorbidities. However, the absence of increased mortality risk suggests autoimmune disease may not independently worsen COVID-19 outcomes. Further study into specific autoimmune conditions and immunosuppressive therapy is needed to guide disease management and improve outcomes for those with autoimmune disease in the context of COVID-19.

**Disclosures:**

Dwayne M. Baxa, PhD, SeaSpine/Orthofix: Grant/Research Support

